# Using imagination in response to stress and uncertainty in the time of COVID-19: further validation of the Fantastic Reality Ability Measurement (FRAME) Scale

**DOI:** 10.3389/fpsyg.2023.1115233

**Published:** 2023-06-15

**Authors:** Dori Rubinstein, Norm O’Rourke, Mooli Lahad

**Affiliations:** ^1^Community Stress Prevention Center, Kiryat Shmona, Israel; ^2^Drama Therapy Graduate Program, Tel Hai Academic College, Kiryat Shmona, Israel; ^3^School of Public Health and Center for Multidisciplinary Research in Aging, Faculty of Health Sciences, Ben-Gurion University of the Negev, Beer-Sheva, Israel; ^4^Department of Psychology, Ben-Gurion University of the Negev, Beer-Sheva, Israel; ^5^Department of Psychology, Tel Hai Academic College, Kiryat Shmona, Israel

**Keywords:** fantastic reality ability, imagination, stress, resilience, scale validity

## Abstract

Fantastic reality ability (FRA) is defined as the capacity to use imagination in response to stress or trauma. With the emergence of COVID-19 and associated social restrictions, there has been an uptick in imagination use as a coping strategy. This has allowed us to further validate the Fantastic Reality Ability Measurement (FRAME) Scale at this time of stress and uncertainty. Initial exploratory factor analyses (EFA) suggested that FRAME responses are measured by four first-order factors. Using confirmatory factor analyses (CFA), this study set out to confirm this finding and to ascertain if: First-order factors are correlated; or map onto a second or higher-order, fantastic reality ability latent construct. Also, FRAME responses are compared to established scales to demonstrate concurrent and discriminant validity. In accord with previous research and theory, CFA results indicate that each four factors (coping, control, transcendence, playfulness), contribute significantly to the measurement of a higher-order FRA latent construct (*N* = 437 Israeli adults). We also report robust correlations between FRAME and measures of resiliency and imagination abilities of complexity, directedness and frequency. Both adaptive and maladaptive applications of imagination use in relation to stress are discussed with focus on those who could foster resiliency. The FRAME enables one to briefly measure imagination use in response to stress and could serve as part of questionnaire batteries measuring individual differences and clinical research. Future research should assess the stability of this instrument across different populations over extended periods, specifically those at risk for trauma.

## Introduction

In March 2020, the World Health Organization (WHO) declared the coronavirus (COVID-19) outbreak a pandemic, later recommending self-quarantine and other social restrictions (World Health Organization, 2020). Though these accommodations slowed the spread of the virus, they also have required new coping skills, some more effective than others. Disengagement and self-isolation, appear associated with loneliness and other negative outcomes (Hoffart et al., 2020; Kirby et al., 2021). In contrast, positive reappraisal and proactive coping strategies such as, active problem-solving and accommodative coping (e.g., and self-encouragement), predict more positive outcomes (Kirby et al., 2021).

Kar et al. (2021) found that those who avoided thinking about the pandemic or were unsure how to cope, and those struggling reported more symptoms of anxiety and depression. Because many have experienced greater stress and more symptoms of depression and anxiety due to COVID-19 and COVID uncertainty, a public health response was warranted. Previous research has found that forced quarantine results in feelings of detachment, irritability, distress, anxiety and post-traumatic stress (Bai et al., 2004; Taylor et al., 2008; Sprang and Silman, 2013; Davis et al., 2021). With the emergence of COVID-19 and associated social restrictions (e.g., shelter-in-place), there have been reports of an uptick in the use of imagination as a coping strategy, both with adaptive and maladaptive applications (Elidrissi, 2021; Zabelina et al., 2021), similar to findings reported before the pandemic (Taylor et al., 1998).

According to Rubinstein et al. (2021, p. 412), “Fantastic reality ability (FRA) is defined as the capacity to use imagination in response to stress or trauma.” A recent review enumerates the various ways in which imagination and related concepts play in fostering resilience and coping with stress (Rubinstein and Lahad, 2022). For this study, we set out to further validate responses to the Fantastic Reality Ability Measurement Scale (FRAME; Rubinstein et al., 2021) in times of distress anxiety and uncertainty (i.e., global pandemic) in order to broaden understanding of how imagination is used to cope with stress and difficult life circumstances.

The 21-item FRAME is a self-report measure of imagination use in response to stress and trauma. Initial exploratory factor analyses (EFA) suggest that FRAME responses are measured by four first-order factors (Rubinstein et al., 2021). Using confirmatory factor analyses (CFA), we intend to replicate this finding and to ascertain if: (1) First-order factors are correlated; or (2) map onto a second-or higher-order, *fantastic reality ability* latent construct.

For this study, we also compare FRAME responses to established scales measuring theoretically-associated and unrelated constructs to demonstrate concurrent and discriminant validity. The COVID-19 pandemic has provided a unique opportunity to further examine the psychometric properties of FRAME responses.

### Imagination as a coping mechanism

In times of uncertainty, reality supersedes imagination. Yet when ‘reality strikes’, imagination maybe our only defence against stress and uncertainty. During pandemic, imagination can foster coping, well-being and recovery. Although imagination can cause pain and suffering for some (e.g., catastrophizing worst case scenarios); imagination can also foster change, comfort and hope under threat (Sheikh, 2003; Awad, 2017; Kalsched, 2021). Imagine a person sheltered-in-place using imagination to invent a new social platform, write a novel; or a six-year-old who pretends to be a ‘face-masked superhero’ saving the world from the next pandemic. Under continuous stress when reality becomes uncertain, both children and adults turn inward to imaginary worlds where they feel secure, gain control and clarity, play and envision solutions. Fantastic Reality (FR) is that imaginative sphere: “The link between the infinite ability of imagination to create a world, desired or required, and actions taken to solve problems that exist in reality” (Lahad, 2017a,b, p. 30). FR entails the ability to transcend to a fantastic realm where people feel impregnable and safe, and where they can manage, adjust and reconstruct the unbearable reality.

Both children and adults reframe meaning through imaginal coping, using spontaneous expressive forms of imagination, especially in times of stress and uncertainty. Such non-literal fantastic forms of expression stem from the human capacity for *as if* thinking, and provide affective relief and flexibility of meaning (i.e., play, humor, pretense, narrative, ritual, metaphor). Openness to flexible imaginings and playfulness as a means of coping can foster resilience (Clark, 2016). Over the lifespan, imagination can serve a fundamental role as a higher mental function in relation to coping and resilience (Awad, 2017). Nonetheless, further research is required to determine how and under what conditions imagination fosters resilience in response to stress and adversity (Capurso and Ragni, 2016; Rubinstein and Lahad, 2022).

Moreover, there is a paucity of quantitative research and tools measuring imagination in relation to stress and trauma. Valid and reliable instruments are needed to identify the determinants and predictors of imagination abilities (Jankowska and Karwowski, 2015; Jung et al., 2016). The FRAME was devised to clarify the role of imagination in coping with stress and trauma (Rubinstein et al., 2021). The COVID-19 provided an opportunity to measure FRAME responses in the general population coping with uncertainty and pandemic related stress.

### Fantastic reality ability measurement (FRAME)

Initial responses to the 21-item FRAME demonstrate high internal consistency (*α* = 0.88) and good test–retest reliability over 27 weeks (0.60 < *r* < 0.80; Rubinstein et al., 2021). Exploratory factor analyses (EFA) suggest that FRAME responses are composed of four distinct first-order factors, consistent with theory (Lahad, 2000, 2005; Lahad and Leykin, 2012; Rubinstein et al., 2021). These results are in accord with recent research supporting the assertion that imagination is not a singular construct, but instead composed of various interrelated elements (Liang and Chia, 2014; Zabelina and Condon, 2020).

FRAME factors are correlated in expected directions with ego resiliency, playfulness and fantasy proneness, suggesting convergent validity. Yet none are strongly correlated with divergent thinking, dissociation or PTSD symptoms, supporting the discriminant validity of responses (Rubinstein et al., 2021). FRAME playfulness, control, and coping factors are correlated with concepts associated with mental health and resilience (i.e., ego resiliency/playfulness) and psychopathology for the transcendence factor (i.e., dissociation, PTSD symptoms). Moreover, transcendence appears distinct from ego resiliency. Initial findings suggest the “dissociative” factor of transcendence contributes most to the associations between FRA and clinical measures. Questions arise from these associations; how is FRA concomitantly associated with both well-being and psychopathology? What are the adaptive/maladaptive abilities of imagination use during adversity or crises? Perhaps a more nuanced, factorial approach is required to delineate problematic from mental health-enhancing FRA.

This study was undertaken to further assess the psychometric properties of FRAME responses during the COVID-19 pandemic at a time of stress and uncertainty. Our intent was to examine how FRA may contribute to understanding of how people manage under adversity. This includes comparison with established measures of imagination and resiliency in relation to the validity of FRAME responses.

## Methods

### Participants

For this cross-sectional, population based study, we recruited Israeli adults using social media advertising as widely used in social science and mental health research (King et al., 2014). A two-week Facebook campaign was conducted July, 2020 for a total of 6,940 exposers. Data collection occurred after the first COVID-19 lockdown in Israel and before vaccines became available. Two lottery prizes of 500 shekels (₪NIS) were awarded to two randomly selected participants. Non-probability-quota sampling was applied so that the sample better reflected the demographic composition of the country (e.g., age, region). The sole inclusion criteria were 18+ years of age, complete FRAME responses and demographic information. This study received ethical approval from the Institutional Review Board (#7/2021–2) at Tel-Hai College, Kiryat Shemona, Israel.

### Measures/instruments

*Fantastic Reality Ability Measurement* (FRAME; Rubinstein, 2020). The 21-item FRAME measures use of imagination in response to stress and trauma. Responses are provided on a Likert scale ranging from *strongly disagree* (1) to *strongly agree* (7). Initial research reports high internal consistency (*α* = 0.88) and good test–retest reliability over 26–28 weeks (0.60 < *r* < 0.80). Responses demonstrate concurrent validity in relation to ego resiliency, playfulness and fantasy proneness (Rubinstein et al., 2021).

Exploratory factor analyses (EFA) suggest FRAME responses measure four factors: *Coping* (5 items; e.g., “When I encounter difficulties or obstacles, I use my imagination to find alternatives to action and problem solving”); *transcendence* (6 items; e.g., “It happens that I find myself so involved in a fantasy or daydream that it feels as though it were really happening to me”); *playfulness* (6 items; e.g., “I enjoy taking part in social games”); and *control* (4 items; e.g., “I control my imagination and I can imagine anything I want”). Responses showed concurrent and factorial validity as playfulness, control, and coping were correlated with “resilient non-clinical” coefficients (i.e., ego resiliency/playfulness) and the transcendence factor with’clinical” measures’ (i.e., dissociation and PTSD symptoms; Rubinstein et al., 2021).

The original *Connor-Davidson Resilience Scale* (CDRISK; Connor and Davidson, 2003) is a 25-item measure of the ability to cope with adversity. Responses are provided on a Likert scale ranging from *not true at all* (0) to *true nearly all the time* (4). The brief CD-RISC-10 is a 10-item version of the CDRISK that measures hardiness, flexibility, sense of self-efficacy, ability to regulate emotion, optimism and cognitive focus/maintaining attention under stress (CDRISK-10; Campbell-Sills and Stein, 2007). The psychometric properties of CDRISK and CDRISK-10 responses have been broadly supported across settings and populations (Ye et al., 2017; Almeida et al., 2020), languages and cultures (Cheng et al., 2020; Chen et al., 2021; Wollny and Jacobs, 2021).

*Four-Factor Imagination Scale* (FFIS; Zabelina and Condon, 2020) is a 26-item instrument with responses reported on a Likert-type scale ranging from *very inaccurate* (1) to *very accurate* (6). The four factors measure *frequency* – the amount of time spent in imaginative states (e.g., “I find myself lost in imagination very frequently”), *complexity* – the detail with which one imagines (e.g., “My fantasies are less detailed than most people’s”), *emotional valence* – the degree to which one’s imagination tends to be positively or negatively valanced (e.g., “I visualize negative outcomes for the future of the world”), and *directedness* – a measure of how goal-oriented (vs. “free-floating”) one’s imagination tends to be (e.g., “My daydreams are directed towards a specific outcome”). Participants are asked to indicate how accurately each statement described them. FFIS responses show good internal consistency, *α* = 0.87 (0.75 < α < 0.94 across subscales), and convergent and discriminant validity relative to established scales measuring related and unrelated constructs, respectively (Zabelina and Condon, 2020; Zabelina et al., 2021).

*Sociodemographic and COVID-19 Information*. Participants were asked to provide descriptive information (e.g., age, gender, region). This included education and marital status. We also asked various questions regarding expose to COVID-19 (i.e., recovered), symptoms and impact on health and well-being (i.e., financial, social, mental and physical health).

### Statistical procedures

Confirmatory factor analyses (CFA) were performed to replicate initial EFA findings suggesting that FRAME responses measure four first-order factors. We also set out to determine if these factors simply covary or whether they map onto a second or higher-order FRA latent construct. Three goodness-of-fit-indices are reported to assess the overall fit of CFA models: An incremental, an absolute, and a parsimonious fit index. The comparative fit index (CFI) is an *incremental index* representing the extent to which a hypothesized model is a better fit to data than the null model. Coefficients greater than 0.94 for the CFI indicate good model fit (Byrne, 2016). The standardized root mean square residual (SRMR) is an *absolute index* which represents the standardized difference between observed and predicted correlations within a hypothesized model. Finally, the root mean square error of approximation (RMSEA) is a *parsimony index* which represents the extent to which a hypothesized model fits data relative to the general population. Coefficients less than.055 for the SRMR and RMSEA suggest good model fit (O’Rourke and Hatcher, 2013).

Correlation coefficients were computed to demonstrate convergent and divergent validity of FRAME responses relative to established scales. SPSS version 26 was used to compute descriptive statistics and correlation coefficients. CFA was performed using AMOS 26.0.

## Results

We analyzed responses from 437 adults aged 18–81 years (*M* = 39.21, SD = 15.18). Tables 1, 2 present descriptive statistics for the study sample. Most participants (78%) were women, and married or partnered (55.6%); 33.4% lived in Tel Aviv or vicinity, and 44.3% had completed a postsecondary degree. Responses were received from an initial sample of 544 respondents; 107 were excluded due to missing data (e.g., entire questionnaire skipped); 437 adults met inclusion criteria, provided complete demographic information and FRAME responses.

**Table 1:** Descriptive statistics of study sample (*N* = 437).

	*n*	%
*Gender*		
Male	96	22
Female	341	78
*Marital Status*		
Single	149	34.1
Cohabiting	243	55.6
Divorced/separated	35	8
Widower	10	2.3
*Education*		
Elementary/high school education	150	34.3
Professional education	93	21.3
University	194	44.4
*Region*		
North	143	32.7
Haifa	47	10.8
Tel-Aviv	46	10.6
Central	100	22.9
Jerusalem	40	9.1
Gaza Envelope	7	1.6
South	54	12.3
*Age*		
18–24	80	18.3
25–34	127	29.1
35–44	68	15.6
45–54	83	19
55–64	50	11.4
65+	29	6.6

**Table 2:** Descriptive statistics, study variables, *N* = 437.

	Mean	SD	Range	Skewness	Kurtosis	Cronbach’s α
Fantastic reality ability	90.96	19.64	21–141	−0.24	0.11	0.87
○ control	17.84	5.26	4–28	−0.31	−0.34	0.81
○ transcendence	22.16	8.09	6–42	0.05	−0.54	0.79
○ playfulness	29.71	6.10	6–42	−0.55	0.66	0.72
○ coping	21.23	5.87	5–35	−0.30	−0.08	0.68
CDRISK-10	27.41	7.08	4–40	−0.55	0.48	0.87
FFIS - Emotional-Valence	4.71	1.17	1–6	−1.05	0.60	0.89
FFIS - Complexity	4.22	1.03	1–6	−0.37	−0.18	0.74
FFIS - Directedness	3.39	1.08	1–6	0.21	−0.57	0.70
FFIS - Frequency	2.50	1.12	1–6	0.79	0.15	0.89

CDRISK, Connor-Davidson Resilience Scale, FFIS, Four-Factor Imagination Scale.

Most participants (76%, or 335 of 437) responded to COVID-related questions; of those, 19 or 6.9% reported that they had recovered from COVID-19 and 40.8% reported that 1+ relatives had been infected. Consistent with the rationale for this study, almost half (43.5%) reported feeling anxious about COVID-19 or its consequences. And many reported that COVID-19 had negatively affected their lives; 32.5% financially, 33.7% socially, and 37.6% mentally.

### Confirmatory factor analysis (CFA)

**Figure 1:**
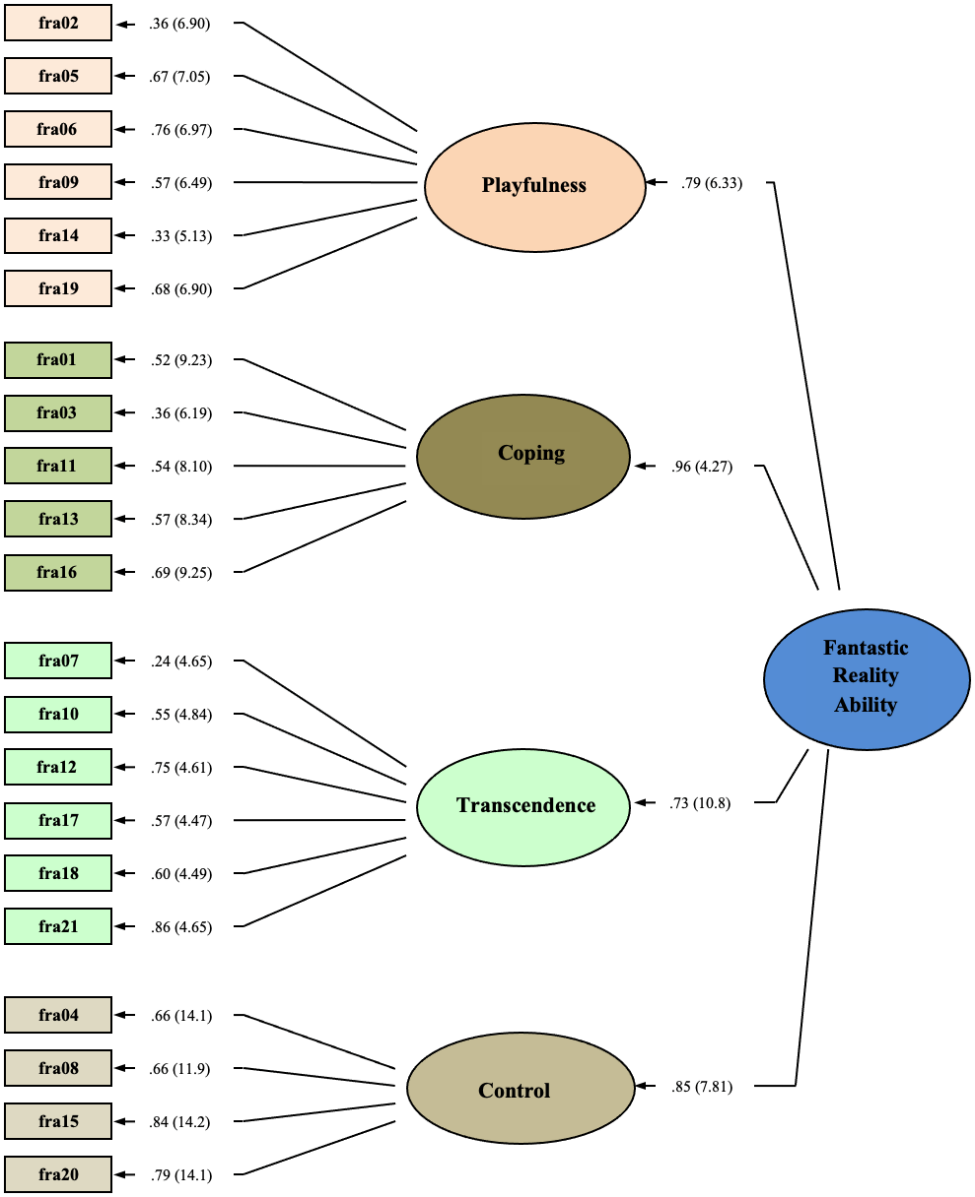
4-Factor, Higher-Order Model, Fantastic Reality Ability. Parameter values expressed as maximum-likelihood estimate standardized solution, χ^2^ = 306.92, *df* = 163, *p* < 0.01, CFI = 0.95, SRMR = 0.047, RMSEA = 0.045 (0.037–0.053). Standardized estimates for FRA items and factors are presented. Parenthetical critical ratio (CR) values represent significance levels of parameter estimates (i.e., all CR > |2.58|, *p* < 0.01).

We performed CFA to replicate and further examine the measurement properties of the 4-factor EFA model (Rubinstein, 2020). As hypothesized, each item significantly measured its respective factor, and each first-order factor significantly measures a second-or higher-order FRA latent construct. CFA was computed using maximum likelihood estimation (Byrne, 2016; see Figure 1).

After correcting for correlated error between 22 of 230 possible pairs, statistics indicated excellent goodness of fit between the higher-order model and data, *χ*^2^ = 306.92, *df* = 163, *p* < 0.01. The comparative fit index is in ideal parameters (i.e., CFI > 0.94, CFI = 0.95), same too for the root mean square error of approximation (RMSEA <0.055, RMSEA = 0.045); the full 90% confidence interval for the RMSEA is in ideal parameters (0.037 < RMSEA CL_90_ < 0.053), as is the standardized root mean residual (SRMR <0.055; SRMR = 0.047). Based on confidence intervals for the RMSEA statistic (Mac Callum et al., 1996), statistical power for this CFA model was estimated at 0.99 (*n* = 437, *df* = 178), sufficient to detect medium to small effects (see O’Rourke and Hatcher, 2013).

### Concurrent validity of FRA responses

To examine convergent and discriminant validity, the FRAME scores were correlated with additional measures administered to participants. Table 3 shows correlations between the FRAME, its four factors, and measures of resiliency and imagination. In general, the FRAME showed small to strong associations with the measures of resiliency (CDRISK-10-Item General Measure). The highest resiliency correlations were with FRAME Playfulness and Control factors (*r* = 0.54 & *r* = 0.46, respectively). Self-Efficacy and Optimism resiliency factors presented most prominently with the FRAME and its subscales (0.29 < *r* < 0.51). FRAME scores (all factors) are moderately correlated with Resiliency (CDRISK-10), Complexity, Directedness and Frequency (FFIS; 0.31 < *r* < 0.44). Consistent with theory, these coefficients suggest concurrent validity of FRAME responses.

**Table 3:** Pearson Correlation Coefficients (*r* values) between FRAME, Resiliency, and Imagination.

Variables	FRA	*Transcendence*	*Playfulness*	*Coping*	*Control*
CDRISK-10 Resiliency	**0.41**		**0.54**	**0.32**	**0.46**
CDRISK-10 Flexibility	0.28		**0.42**	0.20	**0.37**
CDRISK-10 Self-Efficacy	**0.35**		**0.47**	**0.30**	**0.38**
CDRISK-10 Regulate-Emotion	0.25		**0.33**	0.18	**0.31**
CDRISK-10 Optimism	**0.43**	0.13	**0.50**	**0.35**	**0.43**
CDRISK-10 Cognitive-Focus	0.23		**0.36**	0.13	**0.33**
FFIS Emotional-Valence		−0.19	0.24		**0.30**
FFIS Complexity	**0.43**	**0.32**	**0.40**	0.25	**0.36**
FFIS Directedness	**0.32**	0.17	**0.31**	0.28	0.24
FFIS Frequency	**0.36**	**0.65**		0.18	

All *p* < 0.01, *r* > +/−0.30 are bolded to indicate moderate-large associations. CDRISK, Connor-Davidson Resilience Scale, FFIS, Four-Factor Imagination Scale.

Moderate correlation between FRAME factors further suggests construct validity. For instance, Playfulness is strongly correlated with Resiliency (*r* = 0.54) and its subscales (0.32 < *r* < 0.51), and Complexity and Directedness of imagination (*r* = 0.40 & *r* = 0.31, respectively). Control is moderately correlated with Resiliency (*r* = 0.46) and its sub-scales (0.30 < *r* < 0.44), and with imagination Complexity (*r* = 0.36) and Emotional-Valence (*r* = 0.30). Coping is also moderately associated with Resiliency (*r* = 0.32). Finally, Transcendence is strongly associated with imagination Frequency (*r* = 0.65) and moderately associated with imagination Complexity (*r* = 0.32). Consistent with theory, playfulness, control and coping are correlated with ‘resilient’ coefficients. The transcendence factor was mostly correlated with measure of imaginations frequency (*r* = 0.65). These findings provide further support for the concurrent validity of the FRAME and its factors (see Table 3).

### Discriminant validity

FRAME responses are not strongly correlated with the imagination Emotional-Valence (*r* = −0.19). Nor are FRAME responses significantly associated with sociodemographic variables such as age (*r* = −0.08, *ns*), gender (*t* [*df* = 435] = 0.47, *ns*), education (*t* [*df* = 435] = 1.35, *ns*) or region (*t* [*df* = 435] = 0.93, *ns*). The transcendence factor is unrelated to resilience measures.

## Discussion

The results of this study provide further support for the psychometric properties of FRAME responses. As corroborated by participants, the COVID-19 pandemic was a challenging time, suitable to assess how people use imagination in response to stress and uncertainly. In accord with previous research and theory (Lahad, 2000, 2005; Lahad and Leykin, 2012; Rubinstein et al., 2021), CFA analyses supported a four-factor model with each factor contributing to measurement of a second or higher-order FRA latent construct.

The FRAME is a measure of imagination use in response to stress and trauma. Factors measure playfulness, feelings of control when using imagination, imagination as a coping strategy, and the ability to dissociate and transcend into imagination. Participants completed the FRAME during the pandemic as well as associated measures, demonstrating the concurrent and discriminant validity of scale responses. Results of this study support the psychometric properties and factor structure of the FRAME. Moreover, scale responses demonstrate robust correlations with measures of resiliency and imagination complexity, directedness and frequency. These findings are in accord with the operational definition of FRA as an adaptive imagination ability concept (Lahad, 2000, 2005), and previous findings (Rubinstein et al., 2021).

Resilience is strongly correlated with FRAME total scores. Previous COVID-19 research shows that resilient persons use active coping strategies and present with better functioning and fewer symptoms of anxiety and depression (Killgore et al., 2020; Song et al., 2020). FRA may be related to resiliency by using imagination to adapt to situational demands. More research is needed to further understand the relationship between FRA, resiliency and factors mediating or moderating these associations.

The question is, how is imagination used adaptively to contend with stress and uncertainty? In line with theory; control, coping and playfulness are each associated with resilience (i.e., CDRISK-10). These findings are in line with previous work linking ego resiliency and playfulness (Rubinstein et al., 2021). This may suggest that controlled and playful use of imagination as a coping strategy fosters resiliency in times of stress (Rindstedt, 2014; Ferrari, 2016).

Clark (2016) suggests that openness to flexible imagination and playfulness as ongoing coping strategies, can foster resilience. And during the pandemic, Tonkin and Whitaker (2021) described the adaptive value of playfulness on mental and physical health. Perhaps using imagination to cope with stress in a flexible, interactive, social, creative ‘playful’ way, fosters resiliency. Playful individuals report lower levels of perceived stress, more frequent use of adaptive, problem-focused coping strategies and less use of negative, avoidant, and escape-oriented strategies (Magnuson and Barnett, 2013). A recent review discusses how playfulness can foster resilience and coping with trauma (Rubinstein and Lahad, 2022). Clinical research is needed regarding the therapeutic benefits of playful, controlled use of imagination on resilience and coping with stress and trauma.

Associations between the FRAME and FIFS imagination factors (Zabelina and Condon, 2020) may suggest adaptive uses of imagination in times of stress. The “resilient” FRAME factors and especially playfulness appear associated with complexity, and directedness of imagination. One may assume that people using imagination in a playful and controlled fashion, at the same time, use imagination in a goal directed and complex manner. For example, children who initiate imagination into play show concentration abilities, enjoy their activities, develop social and cognitive skills and emotional capabilities, as well as learn to efficiently organize information and effectively integrate external and internal experience (Tower and Singer, 1980; Lieberman, 2014; Møller, 2015). The FRAME control factor is also significantly associated with the emotional-valence of imagination. This is in accord with theory contending that control ability enables use of imagination to foster positive emotions and to regulate emotions.

By corollary, our findings suggest that transcendence as measured by the FRAME is distinct from resiliency. This is in accord with existing research showing that transcendence is unrelated to ego resiliency; rather, transcendence largely accounts for associations between FRA and clinical measures (Rubinstein et al., 2021). Transcendence is instead associated with FIFS frequency and complexity. The ability to detach and become absorbed in imagination during stress and uncertainty appears strongly associated with frequency of imagination use.

Recently Zabelina et al. (2021), reported that frequency of imagination use was associated with higher anxiety during the COVID-19 pandemic, but not before. Moreover, frequency of imagination and loneliness interact, predicting elevated anxiety during (vs. before) the pandemic. These results suggest that at least some features of imagination, particularly frequency, may be associated with negative mood states (e.g., anxiety; Zabelina et al., 2021). Yet other moderating or mediating variables may be in operation (e.g., content, control and playful expression of imagination). Frequent use of imagination may be both adaptive and maladaptive; a wider FRAME factorial map may enhance understanding.

## Clinical implications

Effective measurement of theoretical constructs is paramount for empirical research and clinical application. Use of the FRAME enables clinicians and researchers to assess the various aspects of imagination use before, during, and after exposure to stress and trauma to better understand adaptation and recovery (Zabelina and Condon, 2020; Rubinstein and Lahad, 2022). With the growing demand for focused PTSD treatments (Watkins et al., 2018), the FRAME enables clinicians to determine if specific aspects of imagination are associated with the speed and extent of recovery. Valid and reliable measurement is necessary for comparative research (e.g., randomized controlled trials). This could be done with a range of therapeutic methods that incorporate imagination and play (e.g., mindfulness, SEE FAR CBT, imagery rescripting procedure, Accelerated Resolution Therapy [ART]). Measuring FRA in clinical populations and practice will advance awareness of the role imagination can play in coping and trauma-focused therapies.

## Limitations and direction for future research

The CFA model computed for this study corroborates and extends existing research. After correction for correlated error between 22 of 230 item pairs, goodness of model fit to data is within ideal parameters for each of the indices examined. Though minimal, this adjustment may suggest some items may be redundant or unnecessary. Further psychometric research is warranted to corroborate these findings. An abridged FRAME may demonstrate even better psychometric properties.

Thus far, studies using the FRAME have been conducted in Hebrew, Turkish and German only; research using the English-language version of the scale should be undertaken (Rubinstein et al., 2021) including exploratory and confirmatory factor analyses with other populations. Generalization of findings to other populations may be limited. Longitudinal research is also needed in Israel or abroad with more representative samples in other languages (e.g., Arabic, Russian) including ethnic minorities (e.g., Druse, Bedouin).

Future research should replicate the 4-factor structure with representative samples to establish population norms. Also required is clinical research with trauma-exposed samples and those with trauma-based disorders (e.g., PTSD). Different profiles may be observed with different populations, nature and duration of traumatic exposure, therapeutic interventions, and other coping strategies.

For this study, we further validated FRAME responses during the COVID-19 pandemic. Results corroborate initial findings suggesting that the scale measures four distinct but correlated factors (i.e., coping, control, transcendence and playfulness). We extend this finding to observe that these first-order factors measure a second or higher-order, FRA latent construct. Fantastic reality ability is not a single construct, but a constellation of associated factors.

## Data availability statement

The raw data supporting the conclusions of this article will be made available by the authors, without undue reservation.

## Ethics statement

The studies involving human participants were reviewed and approved by Institutional Review Board (#7/2021–2) at Tel-Hai College, Kiryat Shemona, Israel. The patients/participants provided their written informed consent to participate in this study.

## Author contributions

All authors listed have made a substantial, direct, and intellectual contribution to the work and approved it for publication.

## Acknowledgments

The authors would like to thank Prof. Limor Aharonson-Daniel for her great contribution in the tool development process. Authors are also grateful to David Mizrahi for his assistance with the data collection process in the current study and ongoing support.

## Conflict of interest

The authors declare that the research was conducted in the absence of any commercial or financial relationships that could be construed as a potential conflict of interest.

## Publisher’s note

All claims expressed in this article are solely those of the authors and do not necessarily represent those of their affiliated organizations, or those of the publisher, the editors and the reviewers. Any product that may be evaluated in this article, or claim that may be made by its manufacturer, is not guaranteed or endorsed by the publisher.

## Figures and Tables

**Table:** 

AMOS	Analysis of Movement Structures
CDRISK	Connor-Davidson Resilience Scale
COVID-19	Corona Virus Disease of 2019
CFA	Confirmatory Factor Analysis
CFI	Comparative Fit Index
EFA	Exploratory Factor Analysis
FR	Fantastic Reality
FRA	Fantastic Reality Ability
FRAME	Fantastic Reality Ability Measurement Scale
FFIS	Four-Factor Imagination Scale
PTSD	Posttraumatic Stress Disorder
RMSEA	Root Mean Square Error of Approximation
SD	Standard Deviation
SPSS	Statistical Package for the Social Sciences
SRMR	Standardized Root Mean Square Residual
WHO	World Health Organization

## Appendix

Compendium of acronyms
