# Supplementary material for: Using imagination in response to stress and uncertainty in the time of COVID-19: further validation of the Fantastic Reality Ability Measurement (FRAME) Scale
Source: Front Psychol. 2023 Jun 15;14:1115233. doi: 10.3389/fpsyg.2023.1115233 (PMC10313413; doi:10.3389/fpsyg.2023.1115233)
Supplement: Supplementary file 1 [file Table_1.DOCX]

**Table S1:**

**Table S1 FRA factors correlations coefficients (N = 437).**

| Variables | 1 | 2 | 3 | 4 |
| --- | --- | --- | --- | --- |
| *Transcendence* | - |  |  |  |
| *Playfulness* | .35 | - |  |  |
| *Coping* | .47 | .50 | - |  |
| *Control* | .40 | .58 | .53 | - |

All correlations are at p<.001 level of significance. 95% confidence intervals are between .26 (lower) and .64 (upper).
